# A combination of topical and systemic administration of brimonidine is neuroprotective in the murine optic nerve crush model

**DOI:** 10.1371/journal.pone.0308671

**Published:** 2024-08-08

**Authors:** Ruta Maciulaitiene, Giedrius Kalesnykas, Dainius Haroldas Pauza, Ingrida Januleviciene

**Affiliations:** 1 Department of Ophthalmology, Academy of Medicine, Lithuanian University of Health Sciences, Kaunas, Lithuania; 2 Experimentica Ltd., Kuopio, Finland; 3 Experimentica UAB, Vilnius, Lithuania; 4 Faculty of Medicine and Health Technology, Tampere University, Tampere, Finland; 5 Academy of Medicine, Institute of Anatomy, Lithuanian University of Health Sciences, Kaunas, Lithuania; Bascom Palmer Eye Institute, UNITED STATES OF AMERICA

## Abstract

Glaucoma is a multifactorial optic neuropathy that primarily affecting retinal ganglion cells (RGC). Brimonidine is an intraocular pressure-lowering drug with reported neuroprotective properties. This study aimed to compare the neuroprotective effects of topical and intraperitoneal (IP) brimonidine on RGCs from different retinal segments in a murine optic nerve crush (ONC) model. Methods: forty-one Balb/c mice underwent unilateral ONC and were divided into three study groups: fifteen animals received saline drops twice per day and two additional IP injections of saline; fourteen mice received brimonidine drops twice per day; and 12 mice received brimonidine eye drops twice per day and two additional IP brimonidine injections. Animals were sacrificed seven days post-ONC, and immunohistochemical staining of retinal whole mounts was performed using neuronal NeuN and GFAP staining. Microscopic pictures of the central, middle, and peripheral regions of the retina were taken. The density of the retinal cells was assessed. Results: The total RGC density after ONC and RGC densities in all retinal eccentricities were significantly higher in the brimonidine eye drop and IP combination treatment group than in the saline drop + saline IP, and brimonidine drop treatment groups. Conclusions: brimonidine eye drops supplemented with IP brimonidine injections improved RGC survival in a preclinical model of ONC.

## Introduction

Glaucoma is the most common type of optic neuropathy [1]. Multiple pathogenic mechanisms primarily affect retinal ganglion cells (RGC) [2, 3]. These include the disruption of axonal transport, neurotrophic factor deprivation, metabolic failure, oxidative stress, calcium imbalance, vascular dysregulation, and neuroinflammation. Due to the multifaceted nature of the disease and the limited adaptability of retinal ganglion cells to neurodegenerative changes, glaucoma frequently results in irreversible blindness [4].

In addition to these multiplex neurodegenerative mechanisms, increased intraocular pressure (IOP) and aging are pivotal stressors for RGC in glaucoma [5]. Hence, the primary strategy to treat glaucoma is to lower IOP by decreasing aqueous production and/or promoting aqueous outflow via dedicated conventional or unconventional aqueous outflow pathways [2, 6].

The optic nerve crush (ONC) model has been extensively employed in glaucoma research because of its ability to induce RGC death and axonal degeneration. The ONC model provides a precise framework for investigating traumatic optic neuropathies and the role of axonal damage in glaucomatous pathologies, which mirrors certain human glaucoma characteristics, particularly mechanical stress-induced RGC injury at the optic nerve head [7]. Previous studies have demonstrated that ONC leads to rapid RGC and axon death, with significant decreases in neurite outgrowth parameters and dendritic complexity [8]. The ability of the ONC model to accurately simulate many signaling responses following RGC apoptosis observed in experimental glaucoma models makes it invaluable for elucidating the cellular and molecular mechanisms of glaucomatous diseases.

The topical anti-glaucoma drug brimonidine is an alfa-2 adrenergic receptor agonist that has shown promising results in protecting the retina and optic nerve in non-clinical animal and cell studies [9]. Brimonidine has been suggested to stimulate cell-survival signaling pathways, upregulate neurotrophic factors, and interfere with excitotoxic signaling [10, 11]. The primary mechanism of action is to inhibit adenylate cyclase activity and cyclic adenosine monophosphate (cAMP) levels, thereby decreasing aqueous humor production in the ciliary body [12]. Brimonidine increases aqueous humor outflow due to ciliary muscle relaxation and prostaglandin release due to α-adrenergic stimulation [13]. Brimonidine is known for its strong ability to lower intraocular pressure and its diverse neuroprotective effects [14–17]. These effects include enhancement of neurotrophic factors [10, 18], reduction of cAMP [19, 20], regulation of amyloid beta pathways [21, 22], and modulation of the N-methyl-D-aspartate (NMDA) receptors, which are implicated in glutamate toxicity [21]. Studies have shown that both systemic and topical applications of brimonidine significantly improve the survival of retinal ganglion cells in various rodent models of optic nerve injuries, including acute retinal ischemia [23–25], excitotoxic retinal injury [26], chronic ocular hypertension [27, 28], and optic nerve crush injuries [29, 30].

Furthermore, there is clinical evidence of neuroprotective effects in patients with glaucoma [31], age-related macular degeneration (AMD) [32], retinitis pigmentosa [33], diabetic retinopathy [34], and acute nonarteritic anterior ischemic optic neuropathy [35].

Brimonidine integrated into a polymer-based delivery system has been explored for the treatment of geographic atrophy (GA) associated with AMD [36]. This system exhibited a therapeutic impact lasting up to 15 weeks with encouraging outcomes, indicating a slowdown in the growth of GA lesions.

It has been proven that topical ocular hypotensive drugs delay or prevent glaucoma in patients with ocular hypertension [24, 25]. Nevertheless, subsequent glaucomatous neurodegeneration may occur even when the target IOP is reached. Therefore, it is essential to develop treatments that target neurodegeneration and aid in the preservation of RGC.

Extensive research on the neuroprotective properties of brimonidine has highlighted its potential beyond intraocular pressure reduction, particularly in managing glaucomatous neurodegeneration. This study contributes to the literature by evaluating the effects of topical and systemic administration of brimonidine, including a systemic booster shot approach, in a mouse optic nerve crush (ONC) model.

Specifically, we investigated the regional variations in the survival of retinal ganglion cells and astrocytes across different retinal segments. This approach provides a nuanced understanding of the protective efficacy of brimonidine, potentially enhanced by booster administration, which could lead to more effective targeted therapies to address the complex pathology of optic neuropathies. By integrating comprehensive analyses of drug delivery methods, spatial variability in therapeutic effects, and regional disease variation, this study provides insights that could inform future therapeutic strategies and optimize outcomes in patients with optic nerve injuries.

## Materials and methods

### Experimental design

A total of 41 BALB/c mice were used in this study. An ONC was performed in all mice unilaterally (right eye), and the contralateral eye (left eye) was used as a control group without ONC.

Mice were randomly assigned to one of three experimental groups.

1. Group 1 (ONC/ Saline drop + IP)–mice (n = 15) received saline drops twice daily for seven days. First saline eye drop was instilled on day 0 before ONC. In addition, two doses of 100 μL saline IP injections before ONC at Day 0 and day 1 post-ONC were administered.
2. Group 2 (ONC/BMD drop): mice (n = 14) received topical brimonidine tartrate eye drop treatment twice daily for seven days. First brimonidine eye drop was instilled on day 0 prior to ONC.
3. Group 3 (ONC/BMD drop +IP) mice (n = 12) received topical brimonidine tartrate eye drops twice a day for seven days. First brimonidine eye drop was instilled on day 0 before ONC. In addition, two IP brimonidine injections were administered before ONC on days 0 and 1 post-ONC.

**Fig 1:**
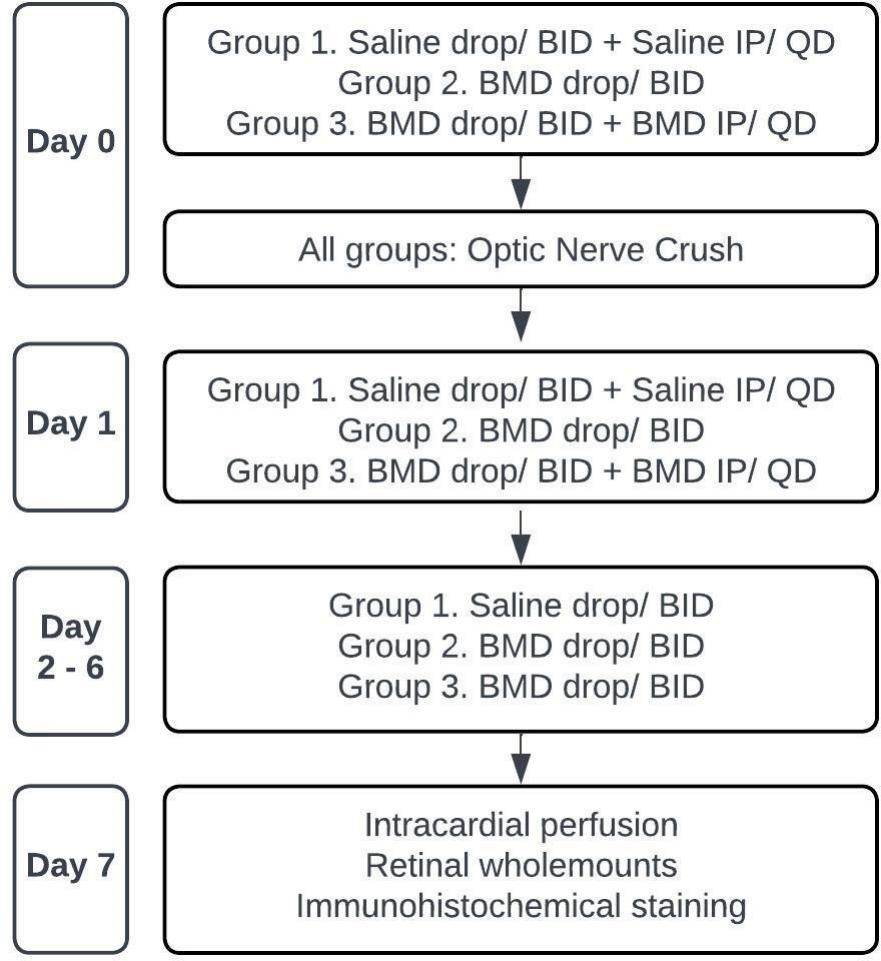
Flowchart of the experimental design. ONC, optic nerve crush; BMD, brimonidine; QD, once daily; BID, twice daily.

Animals were sacrificed on day seven post-ONC, and retinas from both eyes were collected for subsequent analysis. A schematic of the experimental design is shown in Fig 1.

### Animals

All procedures and animal care were performed according to the European Convention of Animal Care and the Association for Research in Vision and Ophthalmology statement for the Use of Animals in Ophthalmic and Vision Research. All experiments were approved by the Lithuanian State Food and Veterinary Service (no. G2-23). Fourty-one healthy 3-month-old male BALB/c/Sca mice were used in this study. The mice were housed in an animal center and maintained under normal light conditions with food and water ad libitum. All procedures were performed under deep intraperitoneal anesthesia using 1 mg/kg medetomidine (Domitor 1 mg/ml; Orion Corporation Pharma, Finland) and 75 mg/kg ketamine (Ketamidor 10%; Richter Pharma AG, Austria). The animals were sacrificed under deep anesthesia by performing intracardial perfusion with aldehyde-based fixatives.

### Mouse model of optic nerve crush

After induction of deep anesthesia, an incision was made in the superolateral conjunctiva to allow gentle outward retraction of the globe using forceps and insertion of the muscle cone. After visual exposition of the optic nerve, self-closing Dumont tweezers were applied for 3 seconds to crush the optic nerve, approximately 2 mm posterior to the globe, according to the methodology described by Kalesnykas et al. [8].

### Brimonidine administration

Mice were treated with brimonidine tartrate ophthalmic solution (Luxfen 2 mg/5 mL, Sanitas, Lithuania) in two forms: topical eye drops twice per day for seven days (one drop (0.05 ml); 2 mg/5 mL, *n* = 14); and two intraperitoneal injections: first IP injection before the ONC, second IP injection on day 1 post-ONC (2 mg/kg/day 2 mg/5 mL brimonidine tartrate, *n* = 12). Group 1 received one saline IP injection before ONC and a second IP injection on day 1 post-ONC (0.1 ml, 0.9% NaCl, *n* = 15), and saline drops (one drop (0.05 ml), 0.9% NaCl) twice a day for seven days.

### Immunohistochemistry and preparation of retinal whole mounts

The mice were euthanized under deep general IP anesthesia seven days after ONC. Soft eye tissue fixation was performed using intracardial perfusion with aldehyde-based fixatives. Both eyes were enucleated and post-fixed in a 4% paraformaldehyde (PFA) solution for 3 h. The retinas of both eyes were detached from the sclera and post-fixed in 4% PFA overnight (O/N). Subsequently, the retinas were washed twice in 0.1 M phosphate buffer solution (PBS) for 5 min. Tissues were incubated in 10% normal goat serum (NGS; Colorado Serum Company, CO) and 0,5% Triton solution (Sigma-Aldrich, St. Louis, MO, USA). Louis, MO, USA) for 30 min. Retinas were subsequently incubated with primary antibodies overnight at 4°C: NeuN (1:500) (MAB377, Millipore Sigma, Massachusetts, USA) and rabbit anti-glial fibrillary acidic protein (GFAP, 1:1000) (Dako (Z0334), Denmark). The retinas were rinsed three times in 1% NGS and 0,1% Triton solution for 5 min and incubated with AlexaFluor Mouse 488 (1:250) (Life Technologies (A11001), California, USA) and Alexa Fluor Rabbit 594 (1:250) (Life Technologies (A11037), California, USA) secondary antibodies for 3 h in the dark. Retinas were rinsed thrice in 1% NGS and 0.1% Triton solution for 5 min, followed by incubation with antibodies. Subsequently, the retinas were incubated with DAPI solution (1:10000) (Sigma-Aldrich (D9542-5NG), Missouri, USA) for 30 min, followed by three rinses in PBS for 5 min. Finally, retinas were mounted on a glass slide using Fluoroshield (Sigma-Aldrich (F6937), Missouri, USA) and covered with coverslips.

### Retinal analysis

The blinding was used for every single analysis. All samples were blinded by a single researcher, and the counting was performed by a lead author of the study.

**Fig 2:**
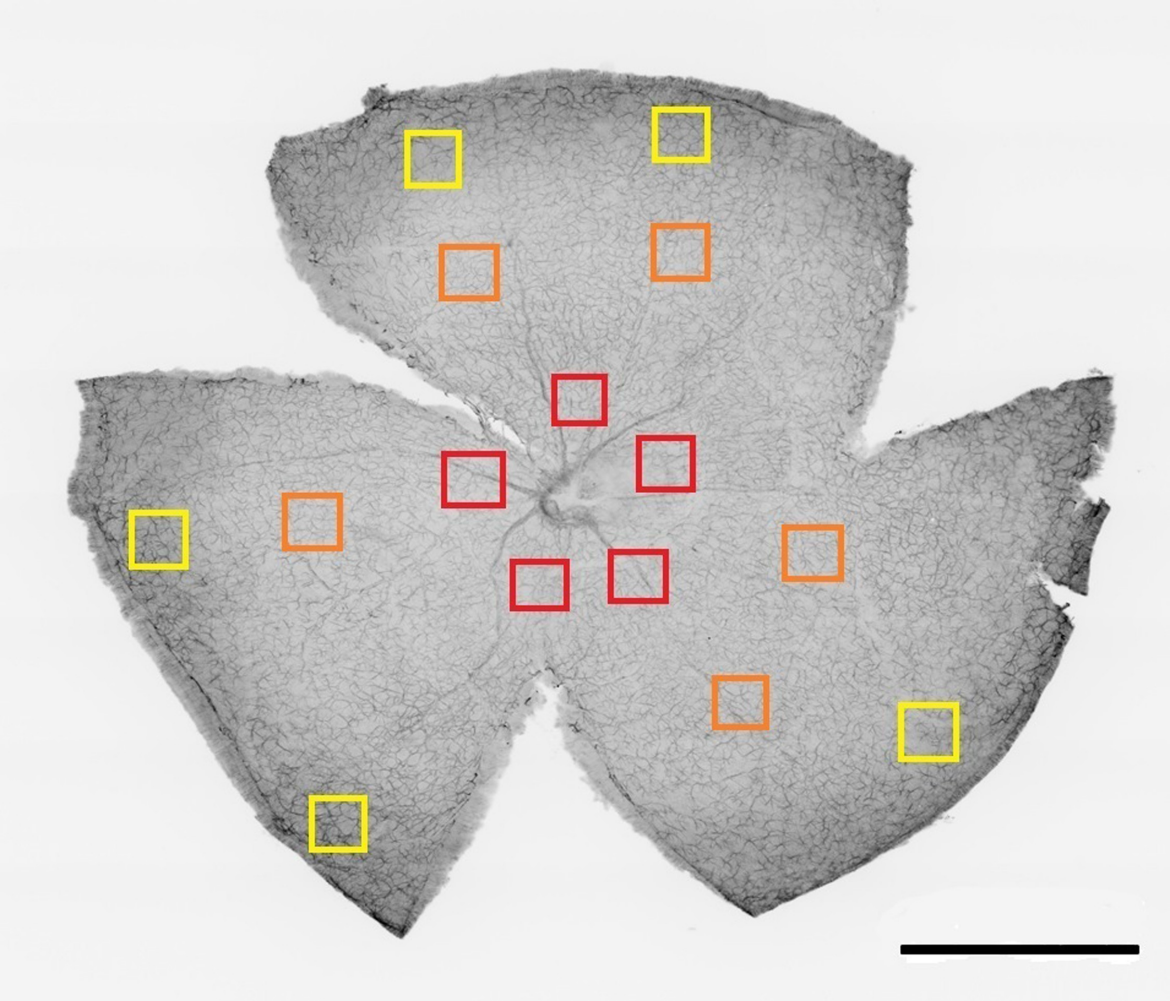
Sampling method for cell counting in retinal whole-mounts. DAPI-, NeuN-, and GFAP-positive cells were counted in 15 randomly chosen sampling boxes (0.04 mm^2^): the central (*n* = 5, red squares), mid-periphery (*n* = 5, orange squares), and peripheral (*n* = 5, yellow squares) regions of the retina. DAPI, 4′,6-diamidino-2-phenylindole; NeuN, neuronal nuclei; GFAP, glial fibrillary acidic protein. Scale bar 1000 μm.

Immunostained whole-mount retinas were analyzed using a fluorescence microscope (Zeiss Axio Imager Z1; Carl Zeiss AG, Jena, Germany) at 40x magnification. Fifteen randomly chosen images of a 0.04 mm2 area were taken from each retina. Five images of each representative central, mid-periphery, and peripheral region of the retina were included for further evaluation (Fig 2). NeuN-, GFAP-, and DAPI-positive cells were manually counted from each image using ImageJ software (Wayne Rasband, National Institutes of Health, USA). The morphology of astrocytes met the criteria of being GFAP-positive cells with cell bodies residing in the nerve fiber layer or ganglion cell layer. DAPI-, NeuN-, and GFAP-positive cells were manually counted in five images from each retinal eccentricity in all wholemounted retinas. To achieve credible results, cells were counted in the entire image, including the left and lower borders. Cells that crossed the right and upper borders were excluded from the analysis.

### Statistical analysis

Quantitative data were analyzed using IBM SPSS (version 26.0; IBM Corporation, USA) and are presented as the mean ± standard deviation (SD).

For ONC analysis, the group averages were calculated by pooling the cell averages. When evaluating dynamic changes, each mouse’s change was calculated individually by comparing the ONC eye with its healthy fellow eye. Individual deltas (Δ) were pooled into groups for comparison.

Parametric data were analyzed using one-way ANOVA variance with Tukey’s multiple comparison test. Non-parametric data were analyzed using the Kruskal-Wallis test. Spearman’s correlation test was used to non-normally distributed data to correlate the variables. Differences were considered statistically significant at *p*≤0.05.

## Results

### Systemic brimonidine side effect

Five out of 12 mice experienced sedative side effects after IP brimonidine injection but regained normal locomotor activity after 60 min.

### Effect of brimonidine treatment on total retinal cells

#### Non-neuronal retinal cells analysis

**Fig 3:**
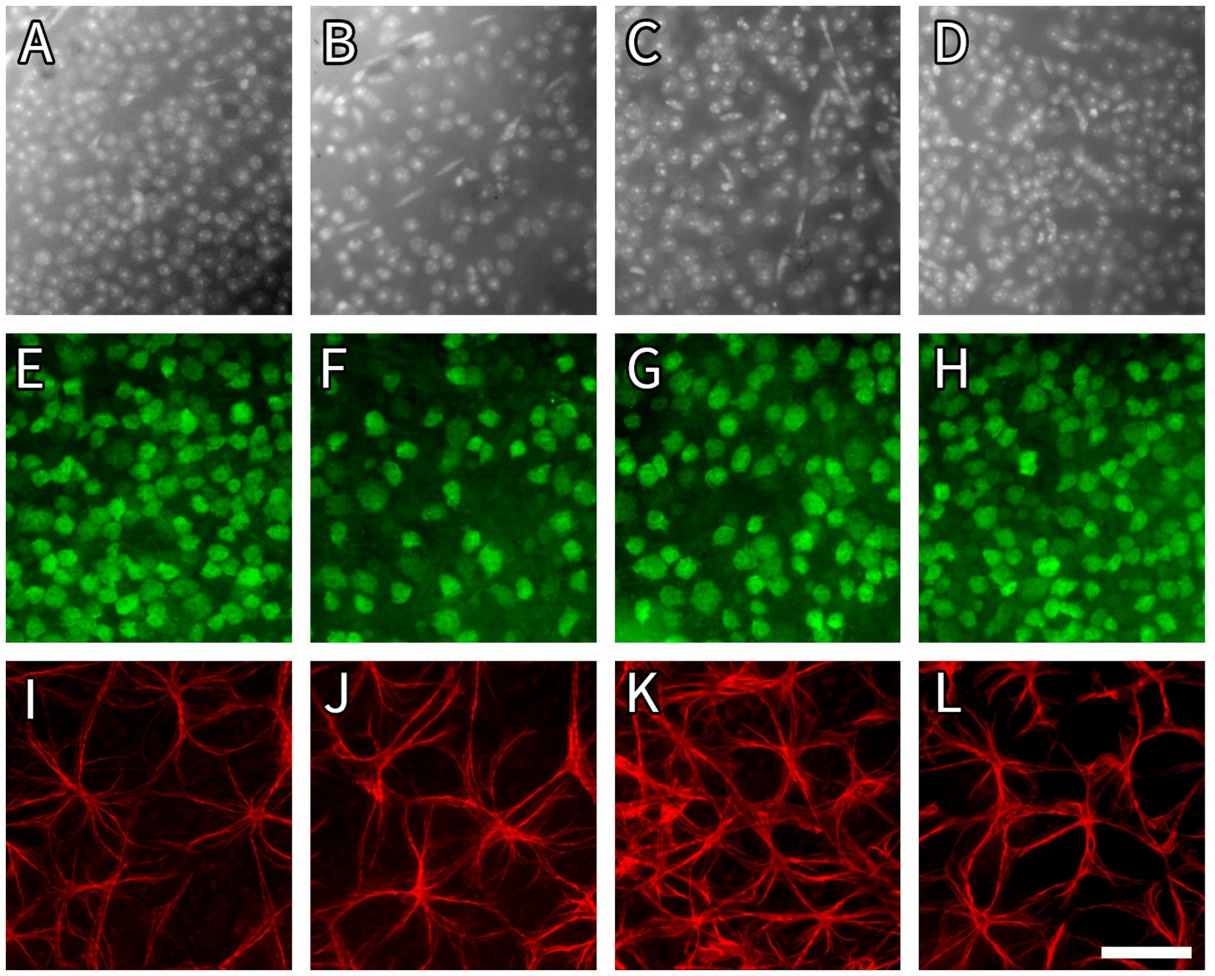
Photomicrograph illustrating immunofluorescence staining of mouse retinal whole-mount in experimental ONC groups, representing retinal cell densities in the experimental ONC and control groups. A, B, C, D: Grey scale DAPI nuclear marker representing all cells of retinal ganglion cell layer; E, F, G, H ‐ NeuN-stained retinal ganglion cells and amacrine cells in green; I, J, K, L–retinal astrocytes (in red) labeled with antibodies against GFAP. A, E, I: retinal whole-mount explan from control group (without ONC); B, F, J: retinal whole mount explant from ONC/saline drop + IP group; C, G, K: retinal whole mount in ONC/BMD drop group; D, H, L: retinal whole mount in ONC/BMD drop + IP group. ONC, optic nerve crush; BMD, brimonidine; IP, intraperitoneal; DAPI, 4′,6-diamidino-2-phenylindole; NeuN, neuronal nuclei; GFAP, glial fibrillary acidic protein. Scale bar– 50 μm.

Optic nerve crush injury was sustained in all the experimental groups. This was evident by the significantly lower DAPI-stained retinal cell count in the ONC/saline drop + IP (6463 ± 1117, cells/mm^2^ ± SD), ONC/BMD drop (6437 ± 915.3, cells/mm^2^ ± SD), and ONC/BMD drop + IP (6691 ± 901.5, cells/mm^2^ ± SD) groups than in the control eyes without ONC (7413 ± 1208, cells/mm^2^ ± SD) (*p*<0.0001). No significant differences were observed between the DAPI-stained cell counts in all the experimental groups after ONC (Fig 3 and S1 Fig and S1 Table).

#### RGC survival analysis

**Fig 4:**
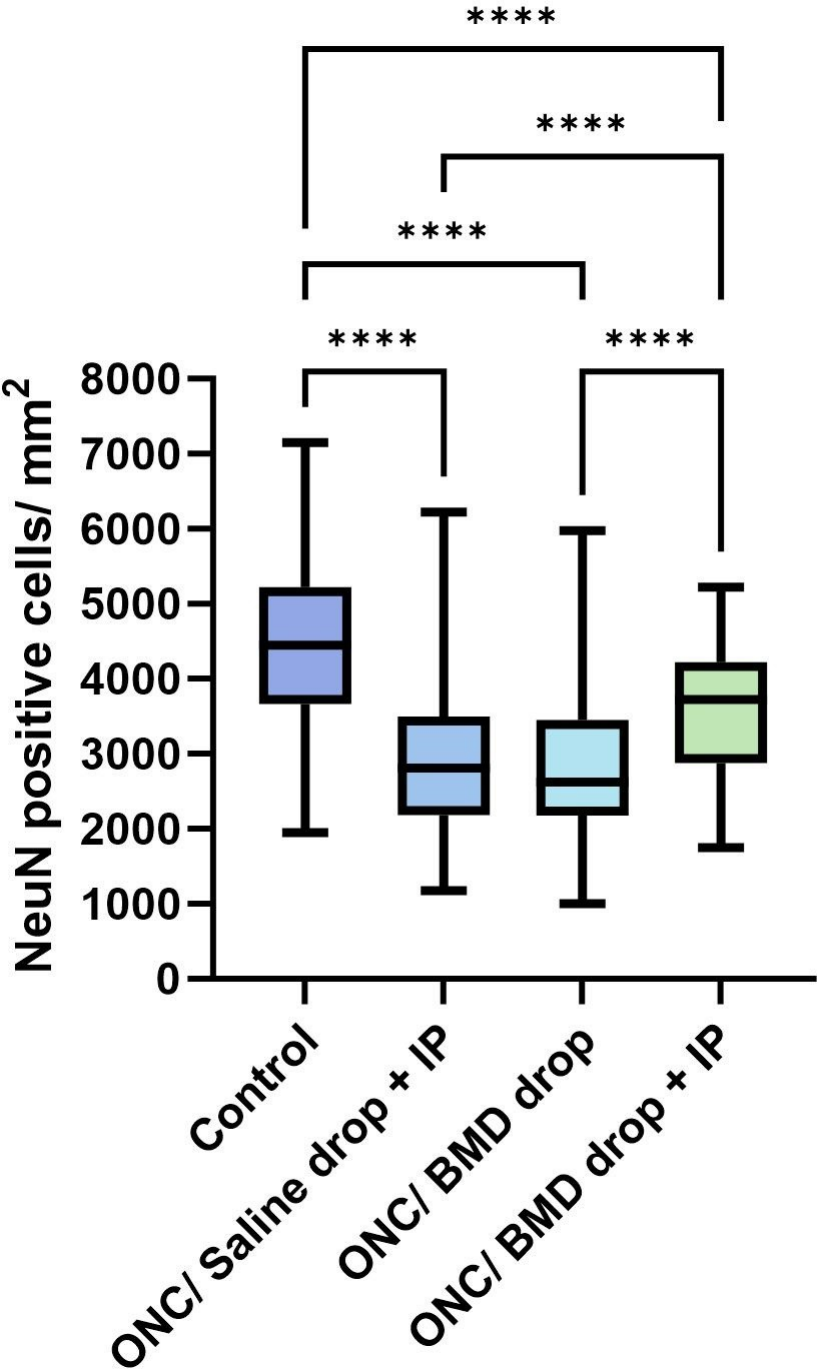
NeuN-stained cell counts in the retina after ONC. **** *p*<0.0001. ONC, optic nerve crush; BMD, brimonidine; IP, intraperitoneal; NeuN, neuronal nuclei.

The remaining NeuN count in the retina was significantly lower in all experimental groups after ONC injury than that in the healthy control group (4450 ± 1140, cells/mm^2^ ± SD) (*p*<0.0001). The additional intraperitoneal administration of brimonidine significantly improved the survival of RGC in the retina (3625 ± 817.9, cells/mm^2^ ± SD) when compared to the ONC/BMD drop (2854 ± 1058, cells/mm^2^ ± SD) and ONC/Saline drop + IP groups (2912 ± 1083, cells/mm^2^ ± SD) (p<0.0001) (Figs 3 and 4 and Table 1).

**Table 1:** NeuN-positive cell count average (cells/mm^2^ ± SD) in the total and different regions of murine retina.

	Total retina	Central region	Middle region	Peripheral region
**Control group**	4450 ± 1140****	4937 ± 1008****	4539 ± 1129***	3911 ± 1049****
**Group 1**	2912 ± 1083	3348 ± 1117	2970 ± 1139	2384 ± 721.4
**Group 2**	2854 ± 1058	2811 ± 1154	3057 ± 1055	2675 ± 968
**Group 3**	3625 ± 817.9****	3505 ± 753.4*	3710 ± 934.5*	3630 ± 737.8***

* *p*<0.05; ** *p*<0.01; *** *p*<0.001; **** *p*<0.0001.

Total retina: **** ‐ Control group vs. Group 1; Control group vs. Group 2; Control group vs. Group 3; Group 3 vs. Group 1; Group 3 vs. Group 2.

Central region: **** ‐ Control group vs. Group 1; Control group vs. Group 2; Control group vs. Group 3; * Group 3 vs. Group 2.

Middle region: *** ‐ Control group vs. Group 1; Control group vs. Group 2; Control group vs. Group 3; * Group 3 vs. Group 1.

Peripheral region. *** ‐ Group 3 vs. Group 1; Group 3 vs. Group 2; **** ‐ Control group vs. Group 1; Control group vs. Group 2

Control group–left eyes without ONC; Group 1 –ONC/Saline drop + IP; Group 2 –ONC/BMD drop; Group 3 –ONC/BMD drop +IP.

ONC, optic nerve crush; BMD, brimonidine; IP, intraperitoneal, NeuN, neuronal nuclei.

#### Retinal astrocytes analysis

Increased glial fibrillary acidic protein (GFAP) immunoreactivity was found in the ONC/BMD drop group (302 ± 79, cells/mm^2^ ± SD) compared to the control group without ONC (278.7 ± 71.13, cells/mm^2^ ± SD) (*p*<0.05). No significant differences were observed in the number of GFAP-positive cells in any of the experimental ONC groups. Interestingly, increased gliosis was evident only in the ONC/BMD drop group (*p* <0.05) (Fig 3 and S2 Fig and S2 Table).

### Regional effect of brimonidine treatment on retinal cells

#### Analysis of the central region of the retina

The survival of DAPI- and NeuN-positive cells in the central region of the retina was comparable to the total cell densities per retina. DAPI- and NeuN-positive cell counts were significantly lower in the ONC/saline drop + IP (6858 ± 1249 and 3348 ± 1117, respectively, cells/mm^2^ ± SD), ONC/BMD drop (6813 ± 807.3 and 2811 ± 1154, respectively, cells/mm^2^ ± SD), and ONC/BMD drop + IP (7004 ± 711.6 and 3505 ± 753.4, respectively, cells/mm^2^ ± SD) groups than in the control group (7930 ± 1133 and 4937 ± 1008, respectively, cells/mm^2^ ± SD) (*p*<0.0001). The additional intraperitoneal administration of brimonidine significantly improved the survival of RGC in the central part of the retina compared to the ONC/BMD drop group (*p*<0.05).

No significant differences were evident between the central region DAPI- and GFAP-positive cells in all experimental ONC groups.

#### Analysis of the middle region of the retina

The resultant remaining cells and their counts in the middle region of the retina resembled the total and central retinal part cell counts.

There were significantly fewer DAPI- and NeuN-stained cells in the middle region of the retina of the control group (7451 ± 1188 and 4539 ± 1129, respectively, cells/mm^2^ ± SD) than in the ONC/BMD drop (6489 ± 863 and 3057 ± 1055, respectively, cells/mm^2^ ± SD) and ONC/BMD drop + IP groups (6649 ± 929.6 and 3710 ± 934.5, respectively, cells/mm^2^ ± SD) (*p*<0.001). Intraperitoneal brimonidine injection significantly improved RGC survival compared to the ONC/saline drop + IP group (*p*<0.05). In addition, a tendency for superior RGC survival was observed in the ONC/BMD drop + IP group compared to the ONC/BMD drop group (*p* = 0.07).

No significant differences were found between the groups in DAPI- and GFAP-positive cells localized to the middle retinal region.

#### Retinal peripheral region analysis

The addition of IP brimonidine injection to brimonidine eye drops had a positive effect on the survival of DAPI-stained cells in the peripheral region of the retina, in contrast to the central and middle regions. This was evident by the insignificant reduction in the DAPI-positive cell count in the ONC/BMD drop + IP group (6390 ± 966.6, cells/mm^2^ ± SD) compared to the control group (6857 ± 1070, cells/mm^2^ ± SD). Markedly decreased DAPI-positive cell counts were found in the peripheral part of the retina in the ONC/saline drop + IP (5954 ± 970.8, cells/mm^2^ ± SD) and ONC/BMD drop groups (6022 ± 905.4, cells/mm^2^ ± SD) compared with the control group (*p*<0.0001). No statistically significant differences were evident between DAPI-positive cells in the peripheral retinal region among all experimental ONC groups.

In addition, the survival of RGCs only numerically declined in the periphery of the ONC/BMD drop + IP group (3630 ± 737.8, cells/mm^2^ ± SD) compared with the control group (3911 ± 1049, cells/mm^2^ ± SD). In contrast, the NeuN-positive cell count in the peripheral region of the retina remained significantly lower in the ONC/saline drop + IP (2384 ± 721.4, cells/mm^2^ ± SD) and ONC/BMD drop groups (2675 ± 968, cells/mm^2^ ± SD) than in the control group (*p*<0.0001).

**Fig 5:**
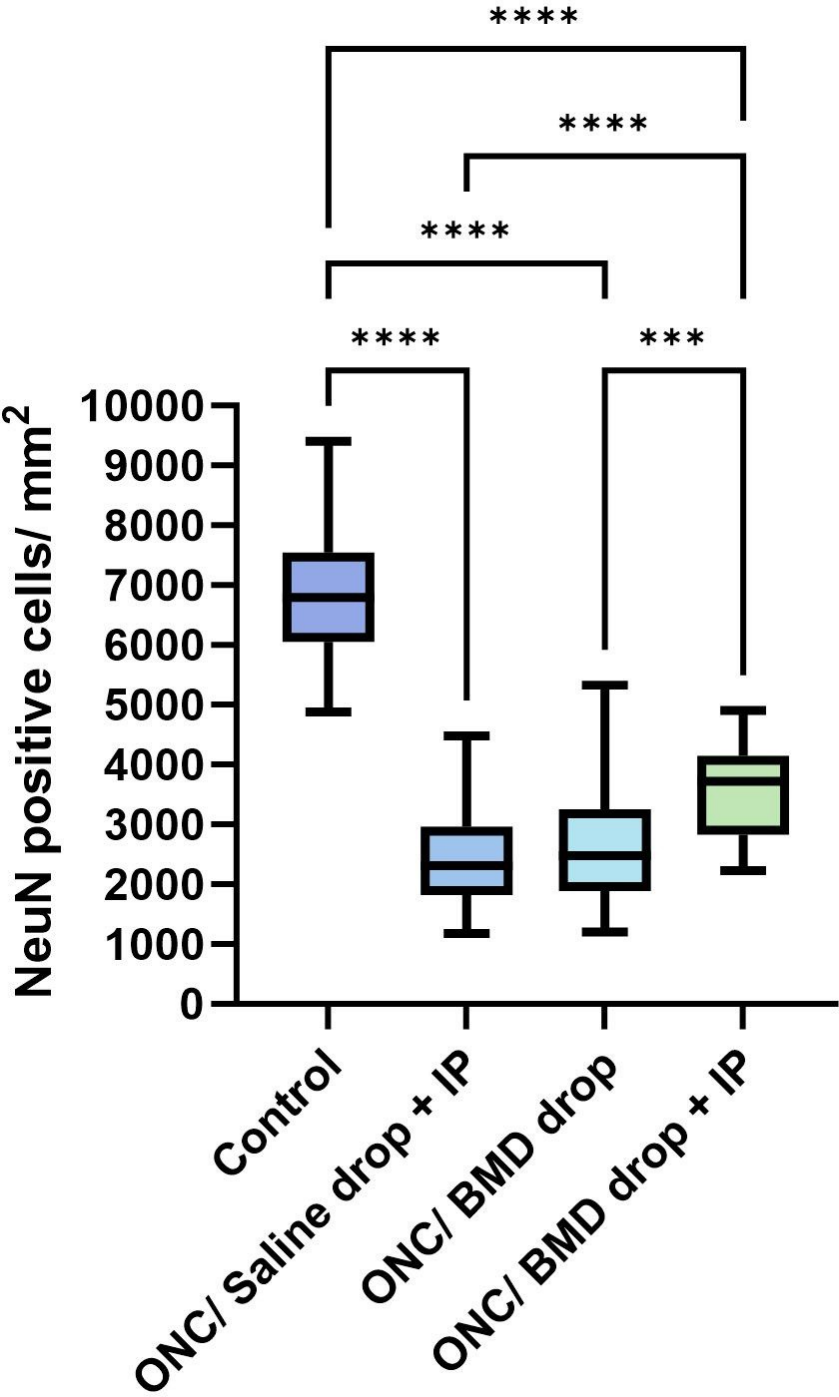
NeuN-stained cell count in the peripheral region of the mouse retina after ONC. ONC, optic nerve crush; BMD, brimonidine; IP, intraperitoneal; NeuN, neuronal nuclei. * *p*<0.05; ** *p*<0.01; *** *p*<0.001; **** *p*<0.0001.

Most importantly, the ONC/BMD drop + IP group showed a positive effect on the NeuN-stained cell count in the peripheral region of the retina. It was significantly higher than that in the ONC/saline drop + IP and ONC/BMD drop groups (*p*<0.001) (Fig 5). No statistically significant differences between peripheral region astrocyte counts in all experimental ONC groups were noted, as evidenced by comparable GFAP staining.

### Regional variability in brimonidine-induced neuroprotection of retinal cells

Analysis of cell count dynamics in the groups treated with brimonidine eye drops revealed a significant difference in DAPI-positive cells between the central and peripheral regions of the retina (*p*<0.05). Furthermore, a markedly significant difference in DAPI-positive density was observed between the middle region and periphery in the ONC/BMD drop + IP group (*p*<0.001).

The superior retention of RGC-positive cells in the peripheral regions of saline-treated groups compared to the central regions (*p*<0.05) underscores the inherent variability in RGC survival after ONC without neuroprotective intervention. Notably, significant differences were also observed in RGC densities when comparing changes between the center and middle (*p*<0.01), as well as between the center and peripheral regions (*p*<0.0001), in groups treated with brimonidine drops alone. The neuroprotective effect of brimonindine when administered systemically, in addition to topical application, further emphasized its neuroprotective capability, as evidenced by the superior retention of RGC densities in the periphery compared to the central part (*p*<0.01) (Table 2).

**Table 2:** Cell count dynamics between retinal regions (Δ cell change ± SD).

Staining method	Retinal region	Group 1	Group 2	Group 3
DAPI	Central retina	1391 ± 730	1711 ± 684.1	1399 ± 652.3
	Middle retina	1165 ± 391.1	1640 ± 566	2034 ± 849.3
	Peripheral retina	1041 ± 504.7	951.6 ± 538.6 *	804.2 ± 452.1 ***
NeuN	Central retina	2272 ± 550 *	2900 ± 760 **,****	2106 ± 962 **
	Middle retina	2088 ± 569.1	1953 ± 609	1748 ± 807.8
	Peripheral retina	1471 ± 587.1	1282 ± 419	871.5 ± 528.8
GFAP	Central retina	-18.16 ± 75.13	-39.47 ± 43.03	-15.17 ± 50.05
	Middle retina	-6.13 ± 26.84	-11.43 ± 36.76	-12.08 ± 29.11
	Peripheral retina	25.87 ± 41.2	0.67 ± 31.67	-0.42 ± 54.15

* *p*<0.05

** p<0.01

*** *p*<0.001

**** *p*<0.0001.

Group 1 –ONC/Saline drop + IP; Group 2 –ONC/BMD drop; Group 3 –ONC/BMD drop +IP. ONC–optic nerve crush; BMD–brimonidine; IP–intraperitoneal, NeuN–primary antibody.

ONC, optic nerve crush; BMD, brimonidine; IP, intraperitoneal; DAPI, 4′,6-diamidino-2-phenylindole; NeuN, neuronal nuclei; GFAP, glial fibrillary acidic protein.

### Correlations between total counts of retinal cells

We analyzed the relationships between DAPI-stained total retinal cell counts, Neun-positive cells, and GFAP expression as indicators of gliosis across different treatment groups.

In the saline-treated groups subjected to ONC, we observed significant correlations between the total number of retinal cells stained with DAPI and GFAP-positive cells (*p*<0.001; *r* = 0.259), indicating a modest direct relationship. Similarly, the correlation between RGC counts and GFAP expression in the ONC/ Saline drop + IP group further suggested a direct but weak association between RGC survival and gliosis without neuroprotective treatment (*p*<0.05, *r* = 0.214). A stronger direct relationship between DAPI and GFAP expression was evident in the ONC/BMD drop group (*p*<0.0001; *r* = 0.285). Conversely, a complex interaction between RGC survival and gliosis when brimonidine was applied topically only was negatively correlated with RGC and astrocyte count in the same group (*p*<0.05, *r* = -0.194) (S3 Fig).

A comparison between DAPI- and NeuN-positive cell counts indicated a direct correlation (*p*<0.01; *r* = 0.315) in the ONC/BMD drop + IP group. This correlation highlights the dependency between non-neuronal cell density and a specific ganglion cell distribution. DAPI was weakly correlated with GFAP in the same group (*p*<0.05; *r* = 0.222) (S3 Fig).

### Intraregional correlations between the retinal cell count

In the central section of the retina, significant correlations were observed between total DAPI-stained retinal cell counts and RGC counts following saline treatment post-ONC (*p*<0.05, *r* = 0.336). This suggests a moderately positive relationship between the non-neuronal cell density and the specific RGC population in the central region of ONC/saline drop + IP group. When comparing the DAPI-stained total retinal cell counts with the RGC counts in the ONC/BMD drop + IP group, a positive correlation was observed (*p* = 0.057, *r* = 0.394), indicating a similar trend in non-neuronal and neuronal cell retention (S4 Fig).

In the middle section of the retina, the correlation between DAPI-stained cells and RGC counts in the ONC/saline drop + IP group was significant (*p*<0.05, *r* = 0.354), revealing a moderately positive relationship between the groups. The ONC/BMD drop + IP treatment group in the middle section showed a nearly significant positive correlation between DAPI-stained total retinal cell counts and RGC counts (*p* = 0.055, *r* = 0.348), and between DAPI-stained cells and GFAP gliosis expression (*p* = 0.059, *r* = 0.297). A significant negative correlation was found between RGC counts and GFAP expression in the ONC/BMD drop treatment group (*p*<0.05, *r* = -0.293) (S4 Fig).

### Interregional correlations between the retinal cell count

A significant correlation was observed between the DAPI-stained cell densities in the central and middle regions of the retina in the ONC/BMD drop group, indicating a moderately positive relationship (*p*<0.001, *r* = 0.447). However, the correlation between the middle and peripheral regions in the ONC/BMD drop + IP group showed a trend towards significance, suggesting a weaker relationship (*p* = 0.054, *r* = 0.319) (S5 Fig).

Strong correlations were found between the retinal eccentricities and RGC densities. The central and middle regions treated with saline drops exhibited a highly significant correlation (*p*<0.0001, *r* = 0.644), which was stronger when comparing the central to peripheral regions (*p*<0.0001, *r* = 0.671) and the middle to peripheral regions (*p*<0.0001, *r* = 0.700). Similarly, under ONC/BMD drop treatment, significant correlations were noted between the central and middle regions (*p*<0.0001, *r* = 0.612) and between the middle and peripheral regions (*p*<0.0001, *r* = 0.601), indicating a consistent pattern of RGC density distribution across the retina post-ONC, irrespective of treatment (S5 Fig).

GFAP expression, which is indicative of gliosis, also showed significant correlations across retinal regions. Central to middle region comparisons in the ONC/saline drop + IP group showed a moderate correlation (*p*<0.05, *r* = 0.282) and a stronger correlation between the central and peripheral regions (*p*<0.01, *r* = 0.342). Notably, positive correlations were more pronounced in the ONC/BMD drop + IP treatment group, with central-to-middle (*p*<0.0001, *r* = 0.578) and central-to-peripheral comparisons (*p*<0.05, *r* = 0.317) (S5 Fig).

### Correlations of retinal cells count dynamics after treatment

A moderate inverse correlation between the incremental change dynamics of GFAP-positive cells and a decrease in NeuN-positive cells in the total retina of the ONC/saline drop + IP group was observed (*p*<0.05, *r* = -0.644). Interestingly, a moderate positive correlation was observed between astrocytes and RGC incremental change in the ONC/BMD drop group (*p*<0.05, *r* = 0.7).

A pronounced positive correlation was observed between the increase in RGC counts and total DAPI-stained cell counts (*p*<0.01, *r* = 0.857) in the middle region of the retinas of the ONC/BMD drop group (S6 Fig).

The negative correlation between changes in RGC counts and GFAP expression in the middle region following saline treatment (*p* = 0.01, *r* = -0.747) underscores a distinct regional interplay, where decreased neuronal survival correlates with increased gliosis.

A comparison of DAPI-stained cell count changes between the middle and peripheral regions following ONC/BMD drop + IP treatment showed a significant correlation (*p*<0.05, *r* = 0.857).

Significant variations were noted in the correlation of the RGC count changes between the peripheral and middle regions after saline treatment (*p* = 0.02, *r* = 0.733), and a similar trend was observed between the middle and central regions after brimonidine eye drop treatment (*p* = 0.057, *r* = 0.714) (S6 Fig).

## Discussion

We investigated the effects of topical administration of brimonidine alone and in combination with IP on the survival of retinal cells in different retinal segments after ONC in a non-clinical murine model. The results suggest that mice receiving combined treatment with topical and systemic brimonidine after ONC showed an overall RGC survival rate of 81,46% in the retina compared to 65,44% RGC survival in the saline treatment group or 64,13% survival rate in the brimonidine eye drop alone treatment group. These results indicate that IP injections of brimonidine supplemented with brimonidine eye drops had an additional benefit for RGC survival after ONC.

Our results are in agreement with those of previous studies, which demonstrated the neuroprotective effects of systemically administered brimonidine. Wheeler et al. showed that local administration of brimonidine eye drops exerts potent neuroprotection and prevents RGC death in a rat model of partial optic nerve crush injury, and that neuroprotection was dose-dependent [11]. Similarly, a systemic brimonidine administration and its effect on the preservation of retinal ganglion cells was evaluated by Lambert et al. [15]. In addition to significantly improved axonal transport and survival of axons, a reduced loss of RGC in the rat retina after 1 mg/kg/day of systemically administered brimonidine has been reported [15]. WoldeMussie et al. studied the effect of 0.5 or 1 mg/kg/d subcutaneous brimonidine administration on RGC survival and compared it with the timolol treatment effect. Their results revealed that brimonidine, but not timolol, has dose-related neuroprotective effects in the retina. Brimonidine had an insignificant effect on IOP if administered subcutaneously [27]. In the study by Marangoz et al., IP administration of brimonidine and melatonin in glaucomatous injury was compared. The results revealed that only 1 mg/kg/day of 0.15% brimonidine had an IOP-lowering effect and acted as a neuroprotective agent in RGCs [16]. Similarly, Hernandez et al. confirmed that once-weekly IP administration of 1 mg/kg brimonidine for 12 weeks increased RGC survival. Additionally, a comparable positive effect was observed after latanoprost. Consequently, the authors assumed that similar neuroprotective results have been achieved via different mechanisms. Inhibition of the apoptotic cascade and reduction of glutamate toxicity have been achieved after IP brimonidine injections, in contrast to latanoprost administration, which resulted in progressively lowered IOP [28].

In summary, most studies that used a 0.5–1 mg/kg dose of IP brimonidine in different regimes [27, 28, 37] from 3 to 12 weeks reported higher RGC survival rates. Our results suggest that a shorter duration of two days but a higher dosage of 2 mg/kg, may have a comparable effect on RGC survival. The observed mild grade 1 adverse events in 5 of 12 mice resolved spontaneously. In addition, the median effective dose (ED50) and lethal dose (LD50) of intraperitoneally administered brimonidine in hypnotized mice were 75.7 mg/kg and the LD50 was 379 mg/kg, respectively [38].

Many studies examining brimonidine’s neuroprotective effects analyze RGCs 1–4 mm from the optic disk or across different retinal regions, averaging these counts to determine mean RGC density [15, 23, 27, 37, 39, 40]. This method may not fully reveal the impact of brimonidine on specific areas because RGC distribution varies across the retina, showing a gradient from the center to the periphery. Research on experimental glaucoma models has noted dense RGCs centrally and sparse RGCs peripherally [28]. Unlike our findings, where elevated IOP led to higher RGC death centrally, previous models showed increased peripheral RGC death due to trabecular blockage and inflammatory responses [41–43]. Hernandez et al. observed consistent RGC survival across the retina following brimonidine treatment under high IOP conditions [28]. Our study contradicts these findings, showing significant central RGC death linked to optic nerve damage, suggesting that injury proximity and model characteristics critically influence RGC survival. This variation across models highlights the importance of further research into the impact of local factors, such as inflammation or mechanical damage, on retinal cell survival.

Our study found that RGCs showed greater survival in the peripheral regions, with a 92.82% survival rate following treatment with brimonidine eye drops and two additional intraperitoneal doses. This survival rate was significantly higher than that with only brimonidine eye drops (68.4%) and the saline control group (60.96%). These results were consistent across the entire retina, confirming the systemic neuroprotective effect of brimonidine.

In addition, studies have highlighted the crucial role of retinal glia in glaucoma, where glial cells support neuronal metabolism and homeostasis under normal conditions [44, 45]. During glaucoma, gliosis occurs as astrocytes and Müller cells react to neuronal damage by becoming inflammatory and contributing to glial scar formation, adversely affecting RGC survival owing to increased pro-inflammatory mediators [46]. Retinal astrocytes, important in ionic balance, neurotransmission, and neurodegeneration, show increased numbers in our brimonidine-treated groups compared to controls, with consistent gliosis across all experimental groups [47–49].

This finding raises questions about the effects of systemic versus topical brimonidine in moderating glial activity. Specifically, whether the addition of IP brimonidine injections could modify the excessive peripheral activation noted, underscoring the complex interplay between systemic and topical treatments in managing gliosis. Interestingly, ONC groups treated with saline drops and IP injections did not show an increase in astrocytes, prompting a reassessment of how various treatments affect glial activation and whether these effects are directly linked to brimonidine’s mechanisms.

In our study examining retinal astrocytes and RGC counts across the retina, we noted a significant inverse correlation in the ONC/BMD drop group, a trend that persisted in the middle retina, indicating that reduced gliosis correlated with higher RGC survival. Conversely, saline treatment showed a direct correlation between astrocyte and RGC counts. Changes in the number of astrocytes in the ONC/saline drop + IP and ONC/BMD drop + IP groups correlated moderately across central to peripheral regions, suggesting controlled gliosis and raising questions about the role of brimonidine in retinal astrogliosis and its dose-dependent effects. Additionally, the ONC/BMD drop + IP group displayed a pronounced protective effect on non-neuronal cells in the peripheral region of the retina, although the difference in DAPI cell counts was not significant compared with the control group. The ONC/saline drop + IP and ONC/BMD drop + IP groups had fewer non-neuronal cells than the control group, suggesting that brimonidine may indirectly benefit both neuronal and non-neuronal cells through systemic and topical application.

Our study has some limitations. One of the limitations of our study was the absence of confirmation of successful application of brimonidine in animals. Ensuring effective delivery and uptake of the treatment would be beneficial for interpreting the neuroprotective outcomes. Additionally, there is a lack of data on the effects of brimonidine treatment on RGCs in the absence of ONC. We cannot entirely rule out the influence of anesthesia on neuroprotection. Nevertheless, all mice were uniformly anesthetized (at the same doses and frequencies), which minimized the potential confounding effects on the results. Further studies are necessary to evaluate the specific impact of brimonidine and anesthesia on RGCs without ONC to better understand their roles in neuroprotection. Using NeuN in conjunction with Brn3a and RBPMS would leverage the strengths of each marker, ensuring a comprehensive assessment of RGC loss and understanding of the broader neuronal context within the ganglion cell layer, enhancing the reliability of our conclusions regarding RGC viability and loss.

In addition, as the main goal of described study was morphological evaluation of RGC and astrocyte survival with an emphasis on possible regional differences of neuroprotective effect of brimonidine, we did not see a viable option to include evaluation of molecular markers from different regions of retina due to the size of sampled tissue and uncertainty on variability. Despite the previously published data confirming the correlation of systemic brimonidine administration and decrease of cAMP [50] and the increase of BDNF [10] in preclinical studies, the study could have benefited from the additional measurement of cAMP or neurotrophic factors to assess the general action of brimonidine in our model.

While we demonstrated the positive impact of additional IP brimonidine injections alongside eye drops on RGC survival, further assessment could benefit from using a pattern electroretinogram to measure functional neuroprotection. Future studies should explore the effects of varying dosages and treatment durations on RGC survival after ONC. Given the observed variations in RGC survival, investigating different glaucoma models may provide deeper insights into treatment efficacy. Each model has unique pathophysiological characteristics that may affect RGC survival differently, underscoring the need for further research across diverse glaucoma paradigms. By examining various dosages and treatment periods across different models, future studies could offer a more detailed understanding of the neuroprotective mechanisms and their broader applicability in managing glaucoma. Such a comprehensive approach would enhance our knowledge of RGC resilience and lead to more effective and tailored treatments for glaucoma.

## Conclusions

IP injections of brimonidine, when used alongside brimonidine eye drops, enhanced RGCs survival across all retinal regions in the mouse ONC model. The reduction in the RGC count was more significant in the central area of the retina. In contrast, gliosis in the retina is predominantly observed in central regions. Our findings indicate that a higher brimonidine dosage of 2 mg/kg or a shorter treatment duration of two days exhibits neuroprotective effects. Further research is necessary to explore the dependency on brimonidine dosage and treatment duration as well as their effects on RGC functional outcomes. Additionally, more detailed studies on the interaction between glial cells and neurons in various segments of the retina would enrich our understanding of neuroprotective mechanisms within the retina.

## Supporting information

S1 Fig DAPI positive cell counts in the total retina after ONC. **** *p*<0.0001. DAPI ‐ 4′,6-diamidino-2-phenylindole–the fluorescent stain; ONC ‐ optic nerve crush; BMD ‐ Brimonidine, IP ‐ intraperitoneal.
(TIFF)

S2 Fig GFAP positive cell counts in the retina after ONC. * *p*<0.05. GFAP ‐ glial fibrillary acidic protein; ONC, optic nerve crush; BMD, Brimonidine, IP, intraperitoneal.
(TIFF)

S3 Fig Correlations between total counts of retinal cells. ONC–optic nerve crush; BMD–brimonidine; IP–intraperitoneal; NeuN–primary antibody; GFAP ‐ glial fibrillary acidic protein; DAPI ‐ 4′,6-diamidino-2-phenylindole–the fluorescent stain; ONC ‐ optic nerve crush; BMD, Brimonidine, IP, intraperitoneal.
(PDF)

S4 Fig Correlations between the regions of the retina. ONC–optic nerve crush; BMD–brimonidine; IP–intraperitoneal; NeuN–primary antibody; GFAP ‐ glial fibrillary acidic protein; DAPI ‐ 4′,6-diamidino-2-phenylindole–the fluorescent stain; ONC ‐ optic nerve crush; BMD, Brimonidine, IP, intraperitoneal.
(PDF)

S5 Fig Correlational analysis of cell density and gliosis across retinal regions. ONC–optic nerve crush; BMD–brimonidine; IP–intraperitoneal; NeuN–primary antibody; GFAP ‐ glial fibrillary acidic protein; DAPI ‐ 4′,6-diamidino-2-phenylindole–the fluorescent stain; ONC ‐ optic nerve crush; BMD, Brimonidine, IP, intraperitoneal.
(PDF)

S6 Fig Correlations of change dynamics. ONC–optic nerve crush; BMD–brimonidine; IP–intraperitoneal; NeuN–primary antibody; GFAP ‐ glial fibrillary acidic protein; DAPI ‐ 4′,6-diamidino-2-phenylindole–the fluorescent stain; ONC ‐ optic nerve crush; BMD, Brimonidine, IP, intraperitoneal.
(PDF)

S1 Table DAPI-positive cell count average (cells/mm^2^ ± SD) in the total and different regions of murine retina. * *p*<0.05; ** *p*<0.01; *** *p*<0.001; **** *p*<0.0001. Total retina: **** ‐ Control group vs. Group 1; Control group vs. Group 2; Control group vs. Group 3; Central region: **** ‐ Control group vs. Group 1; Control group vs. Group 2; Control group vs. Group 3; Middle region: *** ‐ Control group vs. Group 1; Control group vs. Group 2; Control group vs. Group 3; Peripheral region. *** ‐ Control group vs. Group 1; Control group vs. Group 2. Control group–left eyes without ONC; Group 1 –ONC/Saline drop + IP; Group 2 –ONC/BMD drop; Group 3 –ONC/BMD drop +IP. ONC–optic nerve crush; BMD–brimonidine; IP–intraperitoneal, NeuN–primary antibody.
(PDF)

S2 Table GFAP-positive cell count average (cells/mm^2^ ± SD) in the total and different regions of murine retina. * *p*<0.05. Total retina: * ‐ Control group vs. Group 2; Control group–left eyes without ONC; Group 1 –ONC/Saline drop + IP; Group 2 –ONC/BMD drop; Group 3 –ONC/BMD drop +IP. ONC–optic nerve crush; BMD–brimonidine; IP–intraperitoneal, NeuN–primary antibody.
(PDF)
