# Supplementary figures and images for: A combination of topical and systemic administration of brimonidine is neuroprotective in the murine optic nerve crush model
Source: PLoS One. 2024 Aug 8;19(8):e0308671. doi: 10.1371/journal.pone.0308671 (PMC11309405; doi:10.1371/journal.pone.0308671)

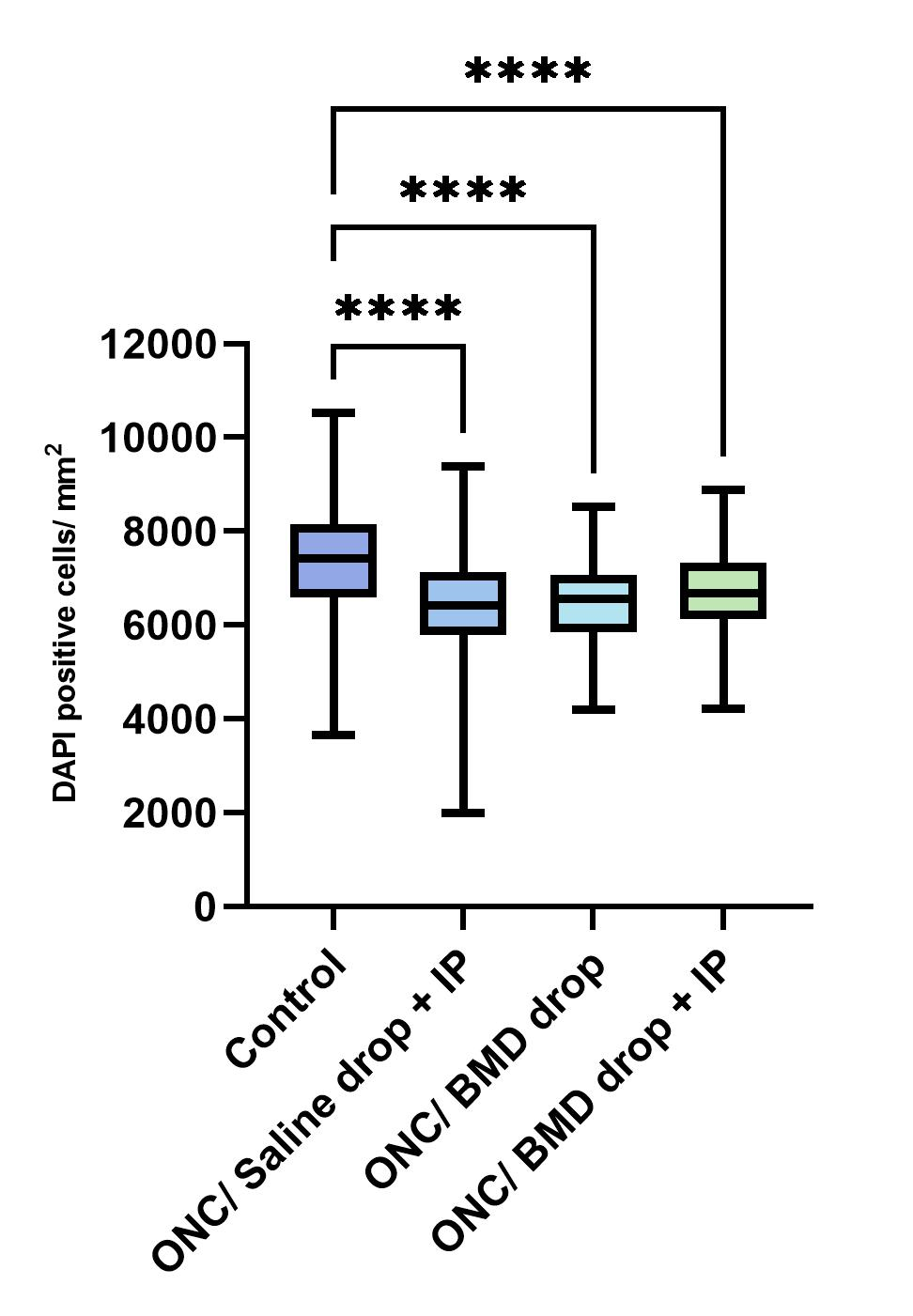

Supplement: S1 Fig — **** p<0.0001. DAPI ‐ 4′,6-diamidino-2-phenylindole–the fluorescent stain; ONC ‐ optic nerve crush; BMD ‐ Brimonidine, IP ‐ intraperitoneal. (TIFF) [file pone.0308671.s001.tiff]

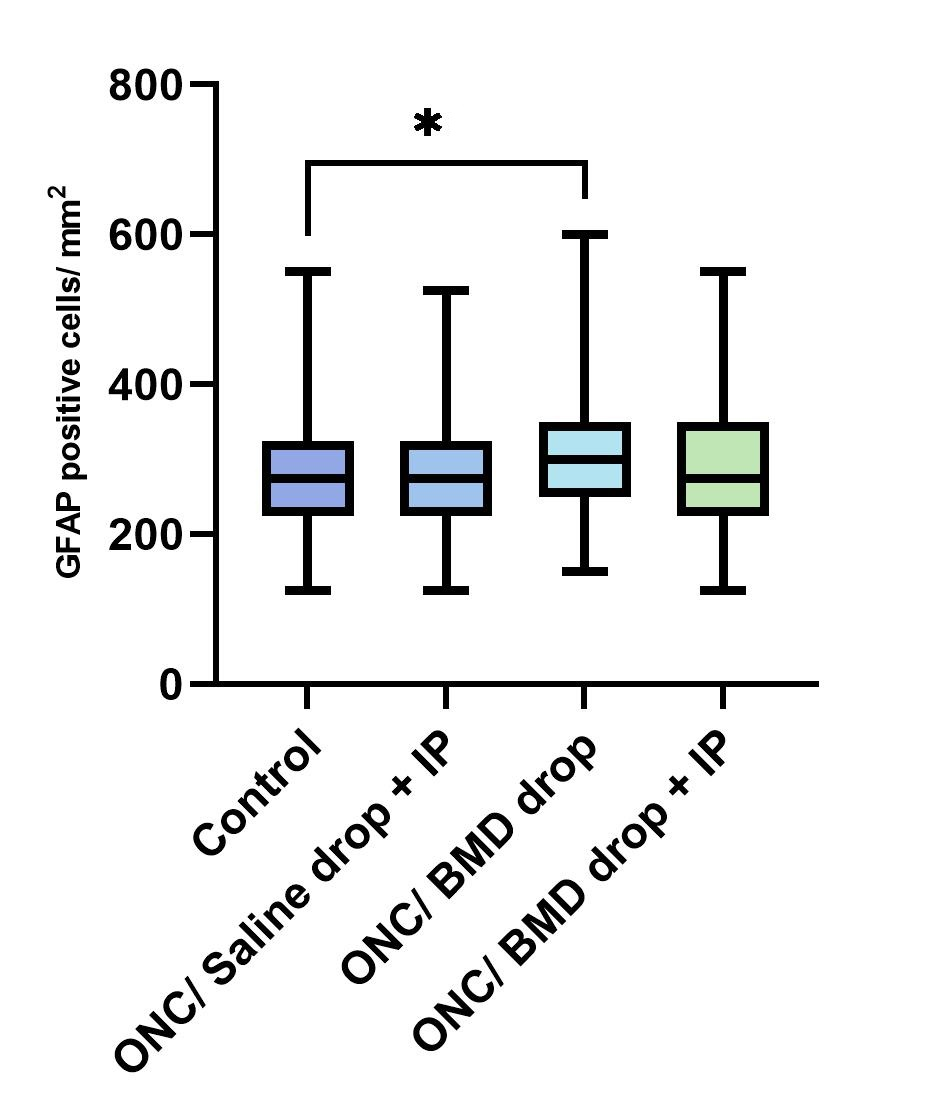

Supplement: S2 Fig — * p<0.05. GFAP ‐ glial fibrillary acidic protein; ONC, optic nerve crush; BMD, Brimonidine, IP, intraperitoneal. (TIFF) [file pone.0308671.s002.tiff]

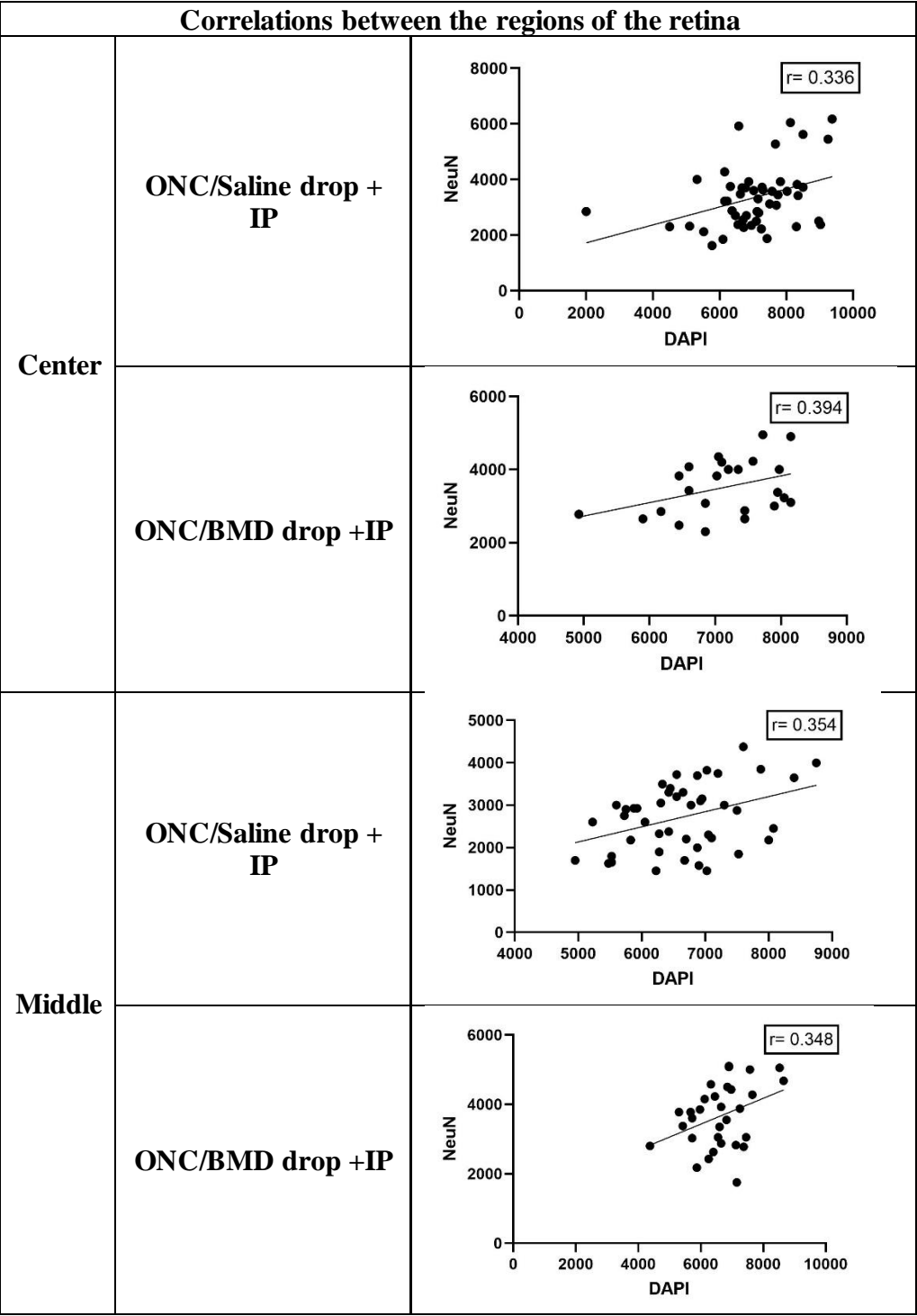

**ONC/BMD drop**

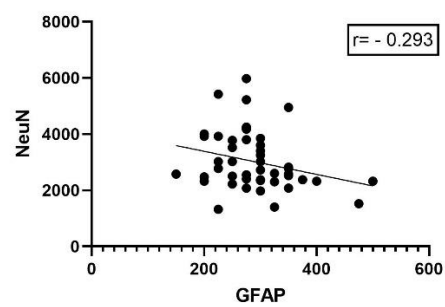

Supplement: S4 Fig — ONC–optic nerve crush; BMD–brimonidine; IP–intraperitoneal; NeuN–primary antibody; GFAP ‐ glial fibrillary acidic protein; DAPI ‐ 4′,6-diamidino-2-phenylindole–the fluorescent stain; ONC ‐ optic nerve crush; BMD, Brimonidine, IP, intraperitoneal. (PDF) [file pone.0308671.s004.pdf]

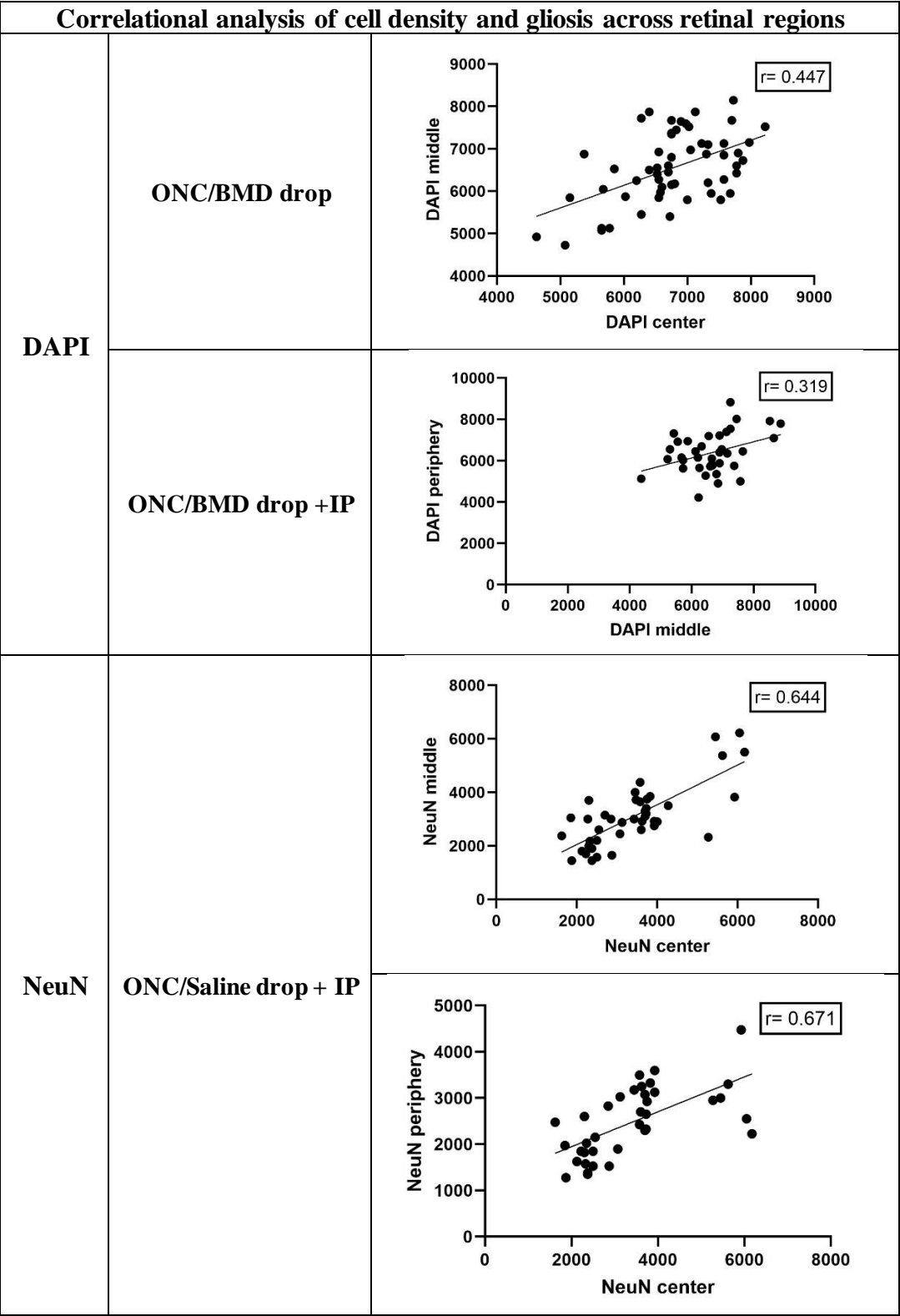

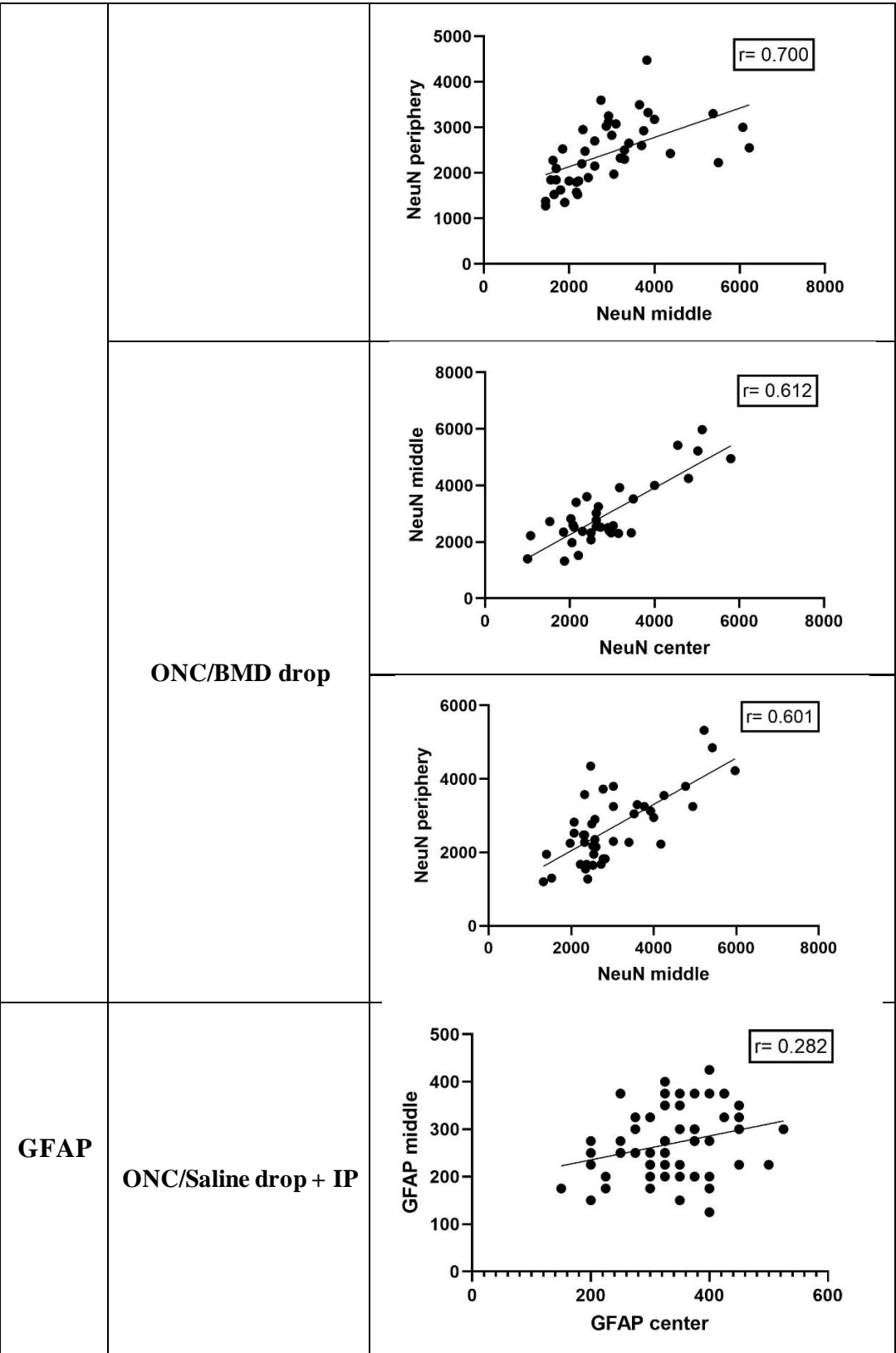

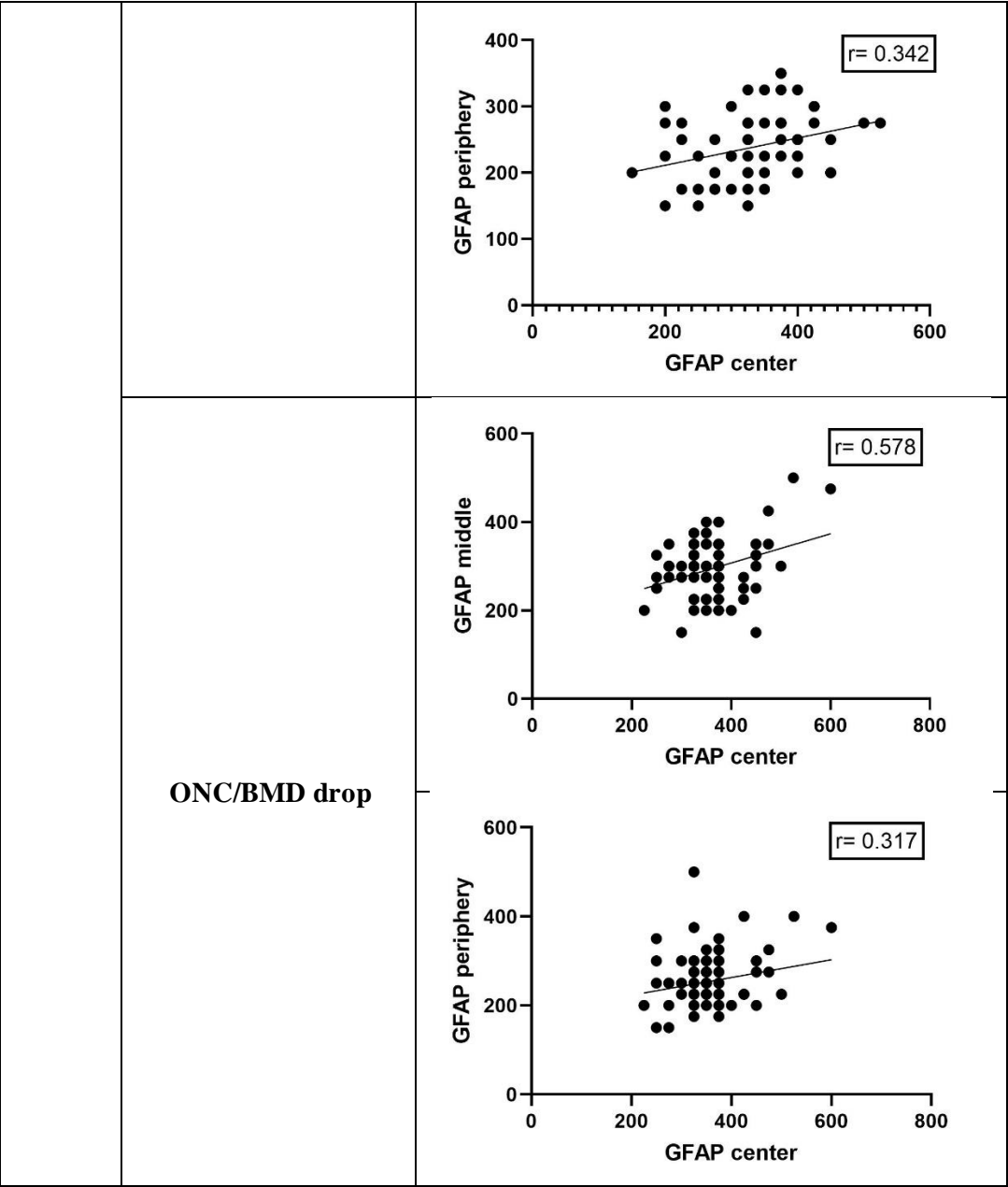

Supplement: S5 Fig — ONC–optic nerve crush; BMD–brimonidine; IP–intraperitoneal; NeuN–primary antibody; GFAP ‐ glial fibrillary acidic protein; DAPI ‐ 4′,6-diamidino-2-phenylindole–the fluorescent stain; ONC ‐ optic nerve crush; BMD, Brimonidine, IP, intraperitoneal. (PDF) [file pone.0308671.s005.pdf]

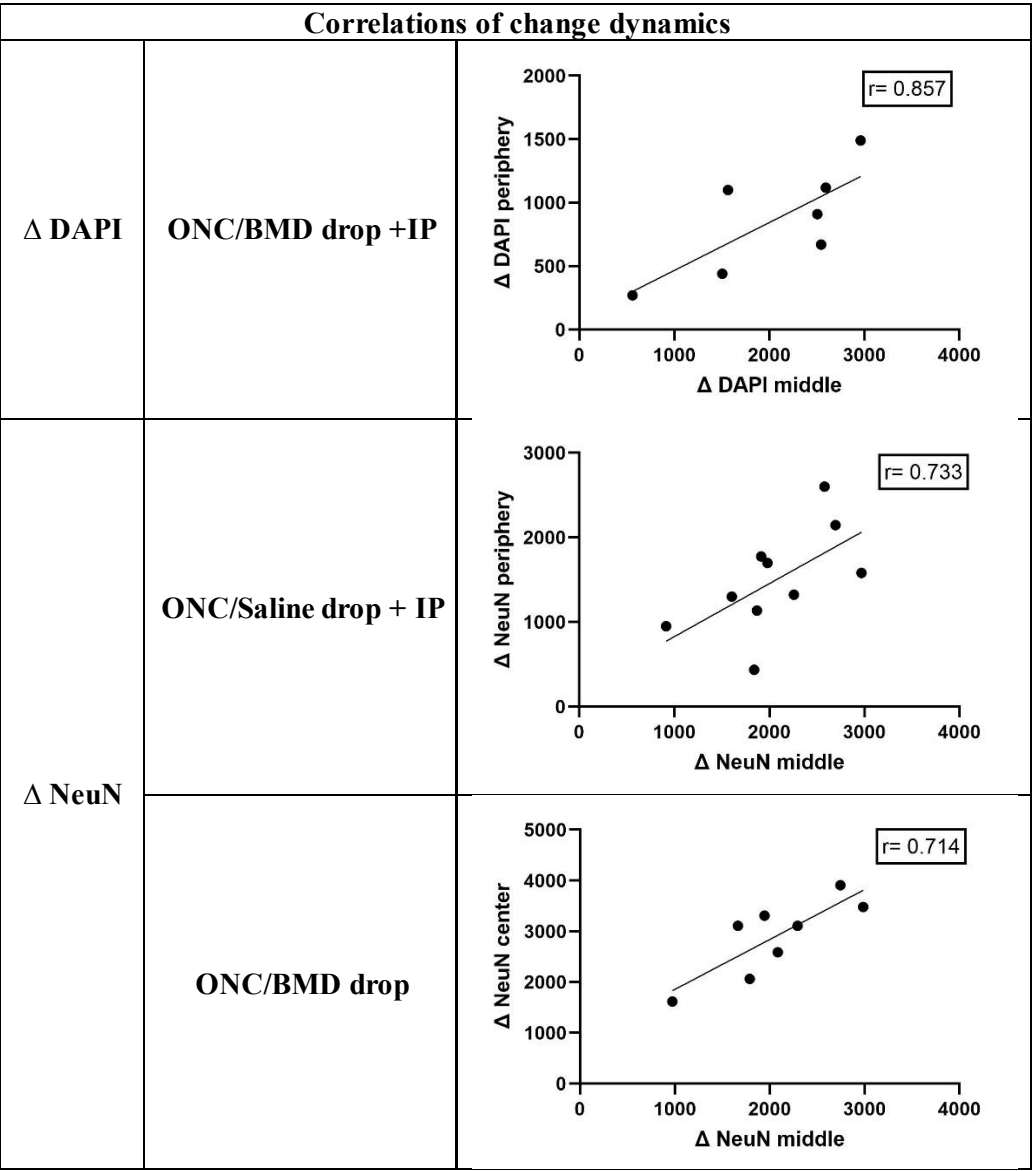

Supplement: S6 Fig — ONC–optic nerve crush; BMD–brimonidine; IP–intraperitoneal; NeuN–primary antibody; GFAP ‐ glial fibrillary acidic protein; DAPI ‐ 4′,6-diamidino-2-phenylindole–the fluorescent stain; ONC ‐ optic nerve crush; BMD, Brimonidine, IP, intraperitoneal. (PDF) [file pone.0308671.s006.pdf]
