# Supplementary material for: A combination of topical and systemic administration of brimonidine is neuroprotective in the murine optic nerve crush model
Source: PLoS One. 2024 Aug 8;19(8):e0308671. doi: 10.1371/journal.pone.0308671 (PMC11309405; doi:10.1371/journal.pone.0308671)

Correlations between total counts of retinal cells

ONC/Saline drop + IP

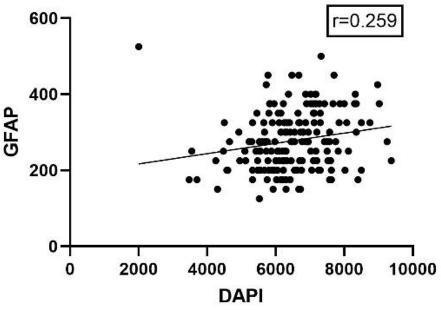

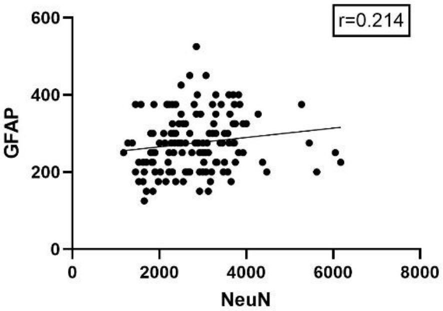

ONC/BMD drop

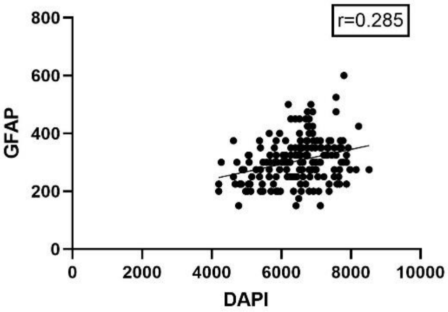

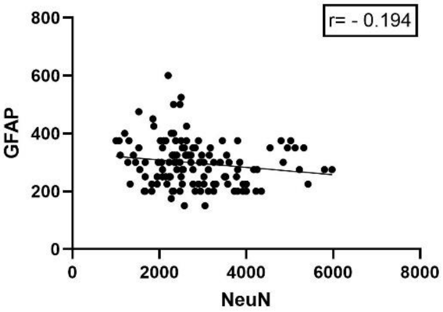

ONC/BMD drop +IP

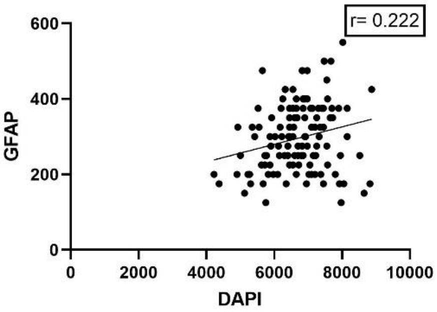

ONC/BMD drop +IP

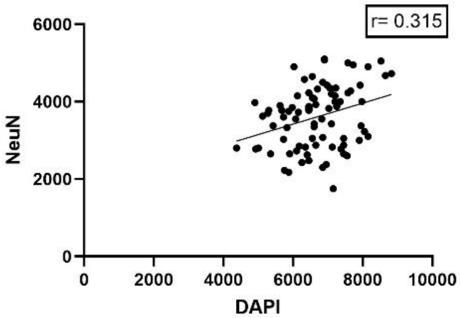

Supplement: S3 Fig — ONC–optic nerve crush; BMD–brimonidine; IP–intraperitoneal; NeuN–primary antibody; GFAP ‐ glial fibrillary acidic protein; DAPI ‐ 4′,6-diamidino-2-phenylindole–the fluorescent stain; ONC ‐ optic nerve crush; BMD, Brimonidine, IP, intraperitoneal. (PDF) [file pone.0308671.s003.pdf]
