# Supplementary material for: A combination of topical and systemic administration of brimonidine is neuroprotective in the murine optic nerve crush model
Source: PLoS One. 2024 Aug 8;19(8):e0308671. doi: 10.1371/journal.pone.0308671 (PMC11309405; doi:10.1371/journal.pone.0308671)
Supplement: S1 Table — * p<0.05; ** p<0.01; *** p<0.001; **** p<0.0001. Total retina: **** ‐ Control group vs. Group 1; Control group vs. Group 2; Control group vs. Group 3; Central region: **** ‐ Control group vs. Group 1; Control group vs. Group 2; Control group vs. Group 3; Middle region: *** ‐ Control group vs. Group 1; Control group vs. Group 2; Control group vs. Group 3; Peripheral region. *** ‐ Control group vs. Group 1; Control group vs. Group 2. Control group–left eyes without ONC; Group 1 –ONC/Saline drop + IP; Group 2 –ONC/BMD drop; Group 3 –ONC/BMD drop +IP. ONC–optic nerve crush; BMD–brimonidine; IP–intraperitoneal, NeuN–primary antibody. (PDF) [file pone.0308671.s007.pdf]

	**Total retina**	**Central region**	**Middle region**	**Peripheral region**
**Control group**	7413 ± 1208****	7930 ± 113****	7451 ± 1188***	6857 ± 1070****
**Group 1**	6463 ± 1117	6858 ± 1249	6559 ± 903	5954 ± 970.8
**Group 2**	6437 ± 915.3	6813 ± 807.3	6489 ± 863	6022 ± 905.4
**Group 3**	6691 ± 901.5	7004 ± 711.	6649 ± 929.6	6390 ± 966.6
