# Supplementary material for: A combination of topical and systemic administration of brimonidine is neuroprotective in the murine optic nerve crush model
Source: PLoS One. 2024 Aug 8;19(8):e0308671. doi: 10.1371/journal.pone.0308671 (PMC11309405; doi:10.1371/journal.pone.0308671)
Supplement: S2 Table — * p<0.05. Total retina: * ‐ Control group vs. Group 2; Control group–left eyes without ONC; Group 1 –ONC/Saline drop + IP; Group 2 –ONC/BMD drop; Group 3 –ONC/BMD drop +IP. ONC–optic nerve crush; BMD–brimonidine; IP–intraperitoneal, NeuN–primary antibody. (PDF) [file pone.0308671.s008.pdf]

	Total retina	Central region	Middle region	Peripheral region
**Control group**	278.7 ± 71.13*	310.3 ± 85.54	263 ± 55.9	262.7 ± 58.26
**Group 1**	280.7 ± 77.15	329.7 ± 77.21	269.3 ± 68.89	238.1 ± 51.17
**Group 2**	302 ± 79	360.5 ± 68.37	293.2 ± 65.99	254.7 ± 63.63
**Group 3**	292.6 ± 85.37	338.4 ± 81.95	290.4 ± 73.95	249.1 ± 77.09
